# Evaluation of Anthropometric Measurements, Arterial Stiffness and ECG Parameters in Alopecia Areata Patients

**DOI:** 10.3390/medicina61122122

**Published:** 2025-11-28

**Authors:** Esra Pancar Yuksel, Gokhan Sahin, Mustafa Cagri Sahin, Sedanur Ozdemir Karadavut, Serkan Yuksel, Fatma Aydin

**Affiliations:** 1 Department of Dermatology, Faculty of Medicine, Ondokuz Mayis University, Samsun 55139, Türkiye; esra.pyuksel@omu.edu.tr (E.P.Y.); sedaozdmr27@gmail.com (S.O.K.); prof.dr.fatmaaydin@gmail.com (F.A.); 2 Samsun Educational and Research Hospital, Samsun 55090, Türkiye; mustafacagrisahin6@gmail.com; 3 Department of Cardiology, Faculty of Medicine, Ondokuz Mayis University, Samsun 55139, Türkiye; serkan.yuksel@omu.edu.tr

**Keywords:** alopecia areata, ECG parameters, anthropometric measurements, body composition, arterial stiffness

## Abstract

*Background and Objectives:* Alopecia areata (AA) is an autoimmune disorder characterized by non-scarring hair loss and has been associated with systemic immune-inflammatory activity and potential cardiometabolic risk. This study aimed to evaluate cardiovascular risk markers—including ECG parameters, arterial stiffness indices, anthropometric measurements, and body composition—in patients with AA according to disease severity and duration, and to compare these findings with healthy controls. *Materials and Methods*: This case–control study included 50 AA patients (32 men, 18 women; mean age: 28.98 ± 9.81 years) and 50 healthy controls (30 men, 20 women; mean age: 28.00 ± 7.86 years). All participants underwent anthropometric and electrocardiographic evaluations. Body composition was assessed, and arterial stiffness was measured. Subgroup analyses were performed according to SALT score (mild vs. moderate-to-severe) and disease duration (≥10 years vs. <10 years). *Results:* Heart rate was lower in AA patients compared with controls (mean difference −5.14 bpm; 95% CI −10.267 to −0.013). No significant differences were found between the groups regarding anthropometric variables, body composition, or arterial stiffness indices. Among AA patients, those with moderate-to-severe disease had significantly lower body fat mass (mean difference 4.95 kg; 95% CI 0.26 to 9.644) and lower visceral fat rating (mean difference 2.428 units; 95% CI 0.800 to 4.056) compared with mild AA. *Conclusions*: AA patients demonstrated lower heart rate and disease-severity-related alterations in body composition, although the clinical significance of these findings remains uncertain. Larger longitudinal studies are needed to clarify whether these subclinical differences translate into meaningful cardiovascular risk over time.

## 1. Introduction

Alopecia areata (AA) is a chronic autoimmune and inflammatory disorder that presents with well-defined hair loss patches without noticeable signs of inflammation or scarring. While the scalp is most commonly affected, hair loss can occur on any part of the body where hair grows [1]. While AA is primarily recognized as a disorder targeting hair follicles, emerging research suggests it may also be linked to broader systemic involvement. Patients with AA have been found to face an increased risk of developing conditions such as atopic dermatitis, metabolic syndrome, lupus erythematosus, iron deficiency anemia, and various thyroid disorders [2]. Moreover, a higher prevalence of coronary artery disease and atrial fibrillation has also been reported in this population [3].

AA is associated with a broad systemic immune-inflammatory activation beyond it is localized cutaneous manifestations. The systemic immune activation and the dysregulation of multiple proinflammatory cytokines that are considered risk factors for developing cardiovascular comorbidities have been reported to be increased in recent studies in AA patients [3,4,5,6,7]. Chronic inflammation and autoimmune dysregulation in AA may trigger endothelial dysfunction and structural vascular changes such as increased arterial stiffness [8].

Noninvasive assessment techniques and different parameters are used to evaluate the cardiovascular risk factors. Electrocardiography (ECG) is one of the noninvasive assessment techniques used to evaluate the heart’s electrical activity. In addition, arterial stiffness is a parameter that refers to the loss of elasticity in the arteries, reported as an important indicator of cardiovascular diseases. Increased waist circumference, hip circumference, and body mass index (BMI) are also reported as risk factors for cardiovascular events [9,10]. So, ECG parameters, anthropometric indices, and arterial stiffness measurements are noninvasive assessment techniques which could be used as early markers of subclinical cardiovascular disease. Alterations in these parameters may precede clinically overt cardiovascular pathology and reflect early endothelial dysfunction, electrophysiological instability, or metabolically driven cardiovascular risk [11]. Therefore, simultaneous assessment of ECG parameters, arterial stiffness indices, and anthropometric markers offers a comprehensive strategy to uncover latent cardiovascular involvement in AA.

Few studies have evaluated non-invasive cardiovascular markers in AA, leaving uncertainty regarding their relationship with disease severity. Chronic inflammatory and autoimmune processes in alopecia areata lead to autonomic imbalance, endothelial dysfunction, and subclinical cardiac involvement. In this study, we hypothesized that AA patients, particularly those with more severe disease, would exhibit abnormal anthropometric parameters, arterial stiffness, and ECG abnormalities.

## 2. Materials and Methods

Fifty AA patients (32 male, 18 female; mean age: 28.98 ± 9.81 years) and 50 healthy controls (30 male, 20 female; mean age: 28.00 ± 7.86 years) were enrolled in this case–control study.

Patients aged 18–65 years with clinically diagnosed AA, alopecia totalis, or alopecia universalis, and who had experienced hair loss for at least one month, were included in the study. Patients with known thyroid, liver, or kidney disease; diabetes; arterial hypertension; dyslipidemia; peripheral or coronary artery disease; a history of myocardial infarction, heart failure, or stroke; autoimmune diseases other than AA; malignancy; pregnancy; breastfeeding; current use of psychiatric medications; or excessive alcohol consumption were excluded. The control group comprised healthy individuals matched for age and gender, with the same exclusion criteria applied.

The diagnosis of alopecia areata was made clinically based on characteristic non-scarring, patchy hair loss, exclamation-mark hairs, and dermoscopic examination was performed when necessary. Their medical histories and current medications as well as disease duration of alopecia areata were recorded. Smoking history was taken and calculated in pack years.

Disease severity in AA patients was determined by calculating the Severity of Alopecia Tool (SALT) score. Disease severity was categorized according to the 2024 European Expert Consensus on systemic treatment of alopecia areata, which classifies AA as mild (SALT 0–20), moderate (SALT 21–49), and severe (SALT ≥ 50) [12]. Based on this consensus, we grouped patients as mild (≤20) versus moderate-to-severe (≥21) AA. This study was approved by the Ondokuz Mayis University Clinical Research Ethics Committee (approval number: 2022/82).

Anthropometric measurements, including waist circumference, hip circumference, and height, were obtained for all participants (patients and controls) using a calibrated tape measure. Weight and body composition parameters—including body fat percentage, fat mass, muscle mass, total body water, total body water percentage, bone mass, and visceral fat level—were measured using the TANITA device (TANITA model 14-2.1-chome, Maeno-cho, Tokyo, Japan). Body composition was assessed under standardized conditions, in a temperature-controlled room (22–24 °C), in a standing position with barefoot, including an 12 h fasting state, avoidance of heavy physical activity, and bladder emptying before assessment.

A 12-lead electrocardiogram (ECG) with standard lead positions was recorded using standard calibrations (10 mm/mV and 25 mm/s). Heart rate, P wave duration and amplitude, P wave dispersion, PR interval, QRS duration, QRS axis, QT interval, QT dispersion, corrected QT interval (QTc), T wave amplitude, and T peak-to-end interval were recorded from the ECGs.

Arterial stiffness was evaluated using the Mobil-O-Graph 24 h ABPM NG^®^ (Stolberg, Germany) arteriography device, with appropriately sized cuffs for each participant. All measurements were obtained from the brachial artery after at least 10 min of seated rest. Participants were instructed to abstain from caffeine, smoking, and exercise for at least 2 h before the assessment. Two consecutive measurements were performed for each participant, and the device-generated averaged pulse wave velocity (PWV) and augmentation index (AIX) values were used for analysis, in accordance with the manufacturer’s guidelines. All assessments were conducted by a single trained operator who was blinded to group allocation and SALT severity. The cuff was inflated to at least 35 mmHg above the measured arterial pressure to occlude brachial artery blood flow fully. Pressure changes resulting in waveforms generated at the membrane level over the brachial artery were detected by sensors within the cuff and transmitted to the device’s tonometer. Using the recorded data, systolic blood pressure, diastolic blood pressure, mean arterial pressure, pulse pressure, AIX, and PWV were calculated via HMS Client Server 5.2^®^ software developed for the arteriography system.

### Statistical Analysis

The required sample size per group was calculated with an alpha level of 0.05 and a statistical power of 80% by using the ClinCalc Sample Size Calculator (Figure S1).

Statistical analyses were conducted using IBM SPSS Statistics version 22.0. Categorical variables were reported as frequencies and percentages. Continuous variables were expressed as the mean ± standard deviation when normally distributed, and as median with minimum and maximum values when they did not meet the assumption of normality. The Kolmogorov–Smirnov and Shapiro–Wilk tests were used to check for normal distribution.

For group comparisons, the independent samples *t*-test was applied to normally distributed data, while the Mann–Whitney U test was used for non-normally distributed variables. The Pearson Chi-square test was employed to compare categorical variables.

Correlation analyses were performed using Pearson’s correlation test when both variables were normally distributed. If normality was not observed in at least one variable, the Spearman rank correlation test was used instead

To evaluate whether alopecia areata (AA) was independently associated with anthropometric, arterial stiffness, and ECG parameters, we performed regression analyses. All regression models were adjusted for potential confounders, including age, sex, body mass index (BMI), smoking (pack years), systolic blood pressure, and diastolic blood pressure. Variance inflation factor values were examined to assess multicollinearity. Results of the regression analyses were reported as unstandardized coefficients (B) with 95% confidence intervals.

A *p*-value < 0.05 was considered statistically significant.

## 3. Results

No statistically significant differences were observed between the patient and control groups in terms of gender (*p* = 0.680) or age (*p* = 0.583). Among the patients, 44 (88%) had alopecia areata, 2 (4%) had alopecia totalis, and 4 (8%) were diagnosed with alopecia universalis. Disease severity, as determined by the SALT score, classified 27 patients as having mild disease and 23 as having moderate to severe disease. The average duration of disease in the AA group was 53.92 ± 68.90 months. There was no significant difference in smoking status between AA patients and controls (χ^2^ = 3.05, *p* = 0.081) (Table S1).

Anthropometric measurements and body composition were compared between the patient and control groups, and no statistically significant differences were observed indicating comparable general body composition profiles (Table 1).

When anthropometric measurements and body composition were compared according to disease severity, AA patients with moderate-to-severe disease demonstrated significantly higher body fat mass (mean difference = 4.95 kg; 95% CI: 0.26–9.644; *p* = 0.036) and higher visceral fat rating (mean difference = 2.428 units; 95% CI: 0.800–4.056; *p* = 0.015) compared with those with mild AA, indicating a severity-related increase in adiposity parameters in Table 2.

A correlation analysis was conducted between the SALT score and anthropometric measurements. A statistically significant, low-to-moderate negative correlation was found between the SALT score and visceral fat level (r= −0.345, *p* = 0.014) (Table S2).

There was also no significant difference between the patient and control groups according to the arterial stiffness findings in Table 3, suggesting no measurable differences in brachial arterial stiffness at the time of assessment.

Among the arterial stiffness parameters, none showed a significant difference between the mild disease group and the moderate-to-severe disease group (*p* > 0.05) (Table S3).

Heart rate was significantly lower in AA patients compared with the controls (mean difference = −5.14 bpm; 95% CI: −10.267 to −0.013; *p* = 0.049), indicating a modest reduction in resting cardiac rate among individuals with AA. No other ECG conduction parameters showed statistically significant differences between the groups (Table 4).

No statistically significant difference was observed in ECG findings between patients with mild and moderate–severe disease (*p* > 0.05) (Table S4).

In the linear regression analysis, AA was independently associated with lower body fat percentage (B = −1.82, 95% CI: −3.35 to −0.29, *p* = 0.020) and lower fat mass (B = −1.57 kg, 95% CI: −2.68 to −0.46, *p* = 0.006), indicating that disease status was linked to modest reductions in adiposity measures even after adjustment for cardiovascular confounders. In contrast, AA showed no independent association with visceral fat (B = −0.28, *p* = 0.398), waist circumference, pulse wave velocity (PWV), augmentation index (AIX), QTc interval, Tp–e interval, or Tp–e/QTc ratio (all *p* > 0.05), suggesting no measurable effects on arterial stiffness or ECG conduction parameters. A summary of the AA effect estimates is presented in Table 5, and detailed outputs of all regression analyses are provided in Tables S5–S13.

## 4. Discussion

This study aimed to explore the differences in ECG, arterial stiffness, and anthropometric parameters between AA patients and healthy controls, as well as to assess the influence of disease severity on these parameters. The study found significant differences in body fat mass in relation to disease severity in AA patients and heart rate in AA patients when compared with healthy controls. However, there are important points to consider when interpreting these results.

In our study, there were no statistically significant differences between the AA group and healthy controls in terms of height, weight, waist circumference, BMI, or visceral fat. These findings suggest that patients with alopecia areata do not exhibit notable obesity or central fat accumulation. Similar results have been reported in previous studies. For example, Wąskiel-Burnat et al. found no significant difference in BMI between AA patients and controls, and Heena Singdia et al. reported comparable waist circumference measurements in both groups [13,14]. In contrast, Conic et al. observed a higher prevalence of obesity and metabolic syndrome in AA patients. However, it should be noted that their study had a smaller sample size compared to other investigations [3]. Therefore, the number of participants should be carefully considered when interpreting and comparing the study results.

Another notable finding of our study is the significantly lower levels of total fat mass and visceral fat in moderate-to-severe AA patients compared to those with mild disease, although there was no difference between the alopecia areata group and healthy controls. Furthermore, a negative correlation was observed between the SALT score and visceral fat level. This indicates that as disease severity increases, there is a corresponding decrease in body fat accumulation. This apparent catabolic process may be explained by several factors such as depression, chronic stress, and appetite suppression, which are possible results of chronic diseases. Besides this, chronic inflammation is the other explanation for adipose tissue loss observed in severe AA [15]. Inflammation due to abnormalities in both innate and adaptive immunity plays a role in adipose remodeling [16]. Macrophages play a central role in this remodeling through the secretion of pro-inflammatory and anti-inflammatory cytokines. However, the relationship between cytokines and adipocytes is complex, and the consequences of adipose tissue inflammation can vary [17]. For instance, paradoxically, TNF-α inhibitors have been reported to cause alopecia in recent years [18]. Considering TNF-α among the inflammatory cytokines and reported to be associated with obesity, future studies about inflammatory cytokines and the underlying mechanisms of adipose tissue inflammation in AA patients may contribute to understanding the relationship between body fat and this disease [17]. However, these mechanisms were not assessed in our study and remain speculative.

Arterial stiffness (AS), commonly measured by the augmentation index (AIx) and pulse wave velocity (PWV), is considered a strong predictor of future cardiovascular events [9]. However, studies examining AS in individuals with alopecia areata (AA) are limited. Najafi et al. identified a positive correlation between PWV and SALT scores, suggesting a potential link between greater disease severity and increased arterial stiffness [6]. Conversely, Waśkiel-Burnat et al. reported no significant differences in AIx values between AA patients and healthy controls, but endothelial dysfunction was observed in 22 out of 52 patients with AA and reported as statistically significant when compared with healthy controls. They concluded regular cardiovascular screening since AA patients showed abnormalities in early predictors of cardiovascular diseases [19]. In our study, we similarly found no statistically significant differences between the AA and control groups in terms of PWV, AIx, or brachial blood pressure measurements. These findings indicate that AA may not be associated with heightened risk for hypertension or increased stiffness of large arteries. Furthermore, disease severity did not appear to affect these parameters. However, the discrepancy between studies may relate to differences in patient age, disease chronicity, comorbidity profiles, measurement devices, and sample sizes, as well as the possibility that AA predominantly affects microvascular or endothelial pathways before macrovessel stiffness becomes detectable. Thus, while our results do not demonstrate increased arterial stiffness in AA, they do not exclude the presence of subtler vascular alterations. Larger longitudinal studies incorporating both macro- and microvascular assessments (e.g., flow-mediated dilation, endothelial biomarkers) are needed to clarify the vascular phenotype of AA.

In our study, heart rate was found to be significantly lower in patients with AA compared to healthy controls. This is consistent with the findings of Peterson et al. [20], who reported a higher prevalence of sinus bradycardia in AA patients based on ECG evaluations. Although the precise mechanism remains unclear, experimental murine models of AA have demonstrated cardiac remodeling, including hypertrophy and increased collagen deposition, which could contribute to conduction abnormalities [21]. Lower heart rate, particularly when accompanied by arterial stiffness or ECG abnormalities, may reflect early manifestations of subclinical cardiovascular involvement in AA. This finding highlights the need for cardiovascular surveillance even in dermatological conditions traditionally considered non-systemic. Identifying reduced heart rate in AA may have clinical implications for risk stratification, as autonomic dysfunction has been shown to precede metabolic disturbances, endothelial dysfunction, and arrhythmogenic vulnerability. Therefore, heart rate assessment could be incorporated into routine evaluation to detect early cardiovascular risk in AA patients [8,9]. Despite this difference in heart rate, other electrocardiographic parameters—T peak-to-end interval/QTc interval, P wave, and QRS duration—did not differ significantly between patients and controls, nor between subgroups based on disease severity. The limited number of studies on cardiovascular complications in AA presents a challenge for drawing direct comparisons. Our results suggest that reduced heart rate may be an isolated finding, and not necessarily indicative of broader cardiac involvement. The lack of significant differences in arterial stiffness and other cardiovascular markers supports this view. However, further research with larger cohorts is needed to better understand potential subclinical or long-term cardiac effects. Future studies are warranted to explore more comprehensive and nuanced markers of cardiac health in this patient population.

This study also has several limitations. Although smoking status did not differ between groups, detailed quantitative assessments of physical activity, alcohol consumption, and socioeconomic factors were not collected, which may contribute to residual confounding. Additionally, inflammatory biomarkers were not measured, limiting the ability to relate cardiovascular findings to systemic immune activity. Subgroup analyses were based on relatively small sample sizes and should therefore be interpreted as exploratory. The disease-duration cut-off was selected for exploratory purposes and requires validation in larger cohorts. Because the analyses were exploratory, no formal correction for multiple comparisons was applied; therefore, marginally significant findings should be interpreted cautiously. Finally, the cross-sectional design precludes causal inference and does not allow for an assessment of the long-term cardiovascular outcomes; thus, the generalizability of the findings to older or higher-risk AA populations is limited.

## 5. Conclusions

This study highlights significant differences in physiological parameters, particularly heart rate, between AA patients and healthy controls, as well as alterations in body composition associated with disease severity. More comprehensive studies are essential to confirm these associations and inform the development of targeted strategies aimed at improving cardiovascular and overall health in individuals living with AA.

## Figures and Tables

**Table 1:** Anthropometric measurements and body composition of patient and control groups.

Parameter	Patient Group (Mean ± SD/Median [Min–Max])	Control Group (Mean ± SD/Median [Min–Max])	*p*-Value
**Height (cm)**	171.36 ± 10.36/171.50 [147–189]	172.44 ± 8.33/173.00 [157–193]	0.567
**Weight (kg)**	71.78 ± 13.12/72.30 [45.00–104.00]	71.42 ± 15.22/71.20 [46.00–113.00]	0.900
**Waist circumference (cm)**	86.10 ± 11.25/85.50 [63.00–114.00]	85.24 ± 12.29/86.00 [65.00–126.00]	0.716
**Hip circumference (cm)**	99.12 ± 8.09/100.00 [82.00–119.00]	99.50 ± 7.54/100.00 [86.00–117.00]	0.809
**Body fat percentage (%)**	20.24 ± 9.52/18.55 [3.70–43.70]	21.39 ± 7.40/21.70 [4.20–39.60]	0.425
**Body fat mass (kg)**	14.62 ± 7.82/14.20 [2.20–41.30]	15.50 ± 6.88/15.25 [2.30–36.40]	0.552
**Muscle mass (kg)**	54.29 ± 10.64/55.65 [35.40–76.10]	53.10 ± 11.15/54.95 [35.10–79.90]	0.588
**Total body water (kg)**	39.97 ± 7.52/41.25 [25.90–54.10]	39.08 ± 7.97/40.10 [25.90–56.40]	0.566
**Total body water percentage (%)**	56.01 ± 6.77/56.80 [39.80–68.50]	54.97 ± 5.08/54.45 [43.80–69.30]	0.387
**Bone mass (kg)**	2.86 ± 0.52/2.90 [1.90–3.90]	2.81 ± 0.55/2.90 [1.90–4.10]	0.618
**Visceral fat level**	4.30 ± 3.25/4.00 [1.00–12.00]	4.18 ± 3.31/3.50 [1.00–15.00]	0.858
**Body mass index (kg/m^2^)**	24.37 ± 3.51/24.00 [18.60–34.70]	23.87 ± 3.97/23.95 [17.00–33.00]	0.508

**Table 2:** Anthropometric measurements and body composition of the patients according to disease severity.

Parameter	Mild Disease (Mean ± SD)	Moderate–Severe Disease (Mean ± SD)	*p*-Value
**Height (cm)**	171.29 ± 10.21	171.53 ± 11.08	0.939
**Weight (kg)**	73.26 ± 14.01	68.31 ± 10.37	0.225
**Waist circumference (cm)**	87.26 ± 12.53	83.40 ± 7.12	0.271
**Hip circumference (cm)**	99.80 ± 8.29	97.53 ± 7.66	0.370
**Body fat percentage (%)**	21.63 ± 9.93	16.28 ± 7.52	0.068
**Body fat mass (kg)**	**16.13 ± 8.33**	**11.11 ± 5.18**	**0.036**
**Muscle mass (kg)**	54.27 ± 11.15	54.35 ± 9.70	0.982
**Total body water percentage (%)**	54.86 ± 7.00	58.69 ± 5.52	0.067
**Bone mass (kg)**	2.87 ± 0.55	2.86 ± 0.47	0.972
**Body mass index (kg/m^2^)**	24.89 ± 3.76	23.17 ± 2.57	0.113
Visceral fat level *	**Mean Rank = 28.73**	**Mean Rank = 17.97**	**0.015**

* Mann–Whitney U test was applied for non-normally distributed variables.

**Table 3:** Arterial Stiffness Parameters in Patient and Control Groups.

Parameter	Patient Group (Mean ± SD/Median [Min–Max])	Control Group (Mean ± SD/Median [Min–Max])	*p*-Value
**Mean systolic blood pressure (mmHg)**	118.44 ± 12.08/117.65 [101.30–180.00]	119.62 ± 10.67/121.10 [96.60–143.30]	0.189
**Mean diastolic blood pressure (mmHg)**	72.16 ± 9.78/71.30 [55.00–105.30]	72.80 ± 10.27/73.65 [49.30–108.00]	0.624
**Mean arterial pressure (mmHg)**	93.17 ± 10.51/93.10 [70.30–139.30]	94.24 ± 9.25/94.95 [73.30–124.00]	0.230
**Mean pulse pressure (mmHg)**	46.23 ± 7.89/45.30 [26.00–74.60]	46.79 ± 9.72/46.45 [28.00–70.30]	0.752
**Mean augmentation index (AIx)**	20.34 ± 10.47/20.65 [–4.00–44.00]	19.33 ± 8.97/19.60 [–4.60–39.00]	0.607
**Mean pulse wave velocity (PWV, m/s)**	5.31 ± 0.84/5.00 [4.30–8.50]	5.24 ± 0.61/5.20 [4.40–6.90]	0.852

**Table 4:** ECG Findings of Patient and Control Groups.

Parameter	Patient Group (Mean ± SD/Median [Min–Max])	Control Group (Mean ± SD/Median [Min–Max])	*p*-Value
**Heart rate (beats/min)**	**73.58 ± 13.96/74.50 (41–102)**	**78.72 ± 11.78/78.00 (56–116)**	**0.049**
**QRS duration (ms)**	89.04 ± 12.21/89.00 (68–113)	87.78 ± 12.38/87.50 (64–114)	0.595
**QT interval (ms)**	363.86 ± 28.77/355.00 (310–444)	354.50 ± 22.31/350.00 (314–408)	0.135
**QTc interval (ms)**	392.52 ± 50.36/400.50 (72–446)	403.02 ± 23.68/402.00 (343–450)	0.306
**PR interval (ms)**	136.86 ± 19.73/135.50 (102–189)	139.90 ± 15.64/138.50 (108–183)	0.395
**P wave duration (ms)**	105.00 ± 23.76/106.50 (46–159)	109.26 ± 16.76/109.00 (80–144)	0.303
**P wave amplitude (mV)**	0.116 ± 0.037/0.100 (0.100–0.200)	0.110 ± 0.030/0.100 (0.100–0.200)	0.375
**T wave amplitude (mV)**	0.450 ± 0.233/0.400 (0.200–1.200)	0.430 ± 0.169/0.400 (0.200–0.900)	0.879
**T peak-to-end interval**	67.72 ± 7.96/70.00 (40–80)	69.20 ± 6.25/70.00 (60–80)	0.481
**P wave dispersion (ms)**	28.90 ± 8.52/30.00 (20–40)	29.40 ± 9.12/30.00 (20–40)	0.790
**QT wave dispersion (ms)**	38.30 ± 13.98/40.00 (20–80)	36.40 ± 12.89/40.00 (10–70)	0.673

Variables were presented as the mean ± standard deviation, median, and minimum–maximum values.

**Table 5:** Multivariable linear regression analyses evaluating the independent association between alopecia areata (AA) and cardiometabolic variables.

Outcome	B (AA)	95% CI	*p*
**Body fat percentage (%)**	−1.819	−3.346 to −0.292	**0.020**
**Body fat mass (kg)**	−1.572	−2.682 to −0.463	**0.006**
**Visceral fat level**	−0.283	−0.944 to 0.378	0.398
**Waist circumference (cm)**	−0.542	−2.699 to 1.615	0.619
**Mean pulse wave velocity (PWV, m/s)**	0.057	−0.012 to 0.126	0.107
**Mean augmentation index (AIx)**	2.024	−1.521 to 5.568	0.260
**QTc interval (ms)**	−10.675	−25.905 to 4.555	0.167
**T peak-to-end interval**	−1.928	−4.705 to 0.848	0.171
**T peak-to-end interval/QTc interval (ms)**	0.012	−0.015 to 0.040	0.372

## Data Availability

The data that support the findings of this study are available from the corresponding author upon reasonable request.

## Supplementary Materials

The following supporting information can be downloaded at: https://www.mdpi.com/article/10.3390/medicina61122122/s1, Figure S1: The power analysis performed for the study; Table S1: Smoking status among AA patients and controls; Table S2. Correlation Between SALT Score and Anthropometric Measurements; Table S3. Arterial Stiffness Findings of the Patients According to Disease Severity; Table S4: ECG Findings of the Patients According to Disease Severity; Table S5. Multivariate regression analysis of body fat percentage (%); Table S6. Multivariate regression analysis of body fat mass (kg); Table S7. Multivariate regression analysis of visceral fat level; Table S8. Multivariate regression analysis of waist circumference (cm); Table S9. Multivariate regression analysis of mean pulse wave velocity (PWV, m/s); Table S10. Multivariate regression analysis of mean augmentation index (AIx); Table S11. Multivariate regression analysis of QTc interval; Table S12. Multivariate regression analysis of T peak-to-end interval; Table S13. Multivariate regression analysis of T peak-to-interval/QTc interval.

## Abbreviations

The following abbreviations are used in this manuscript:

AA	Alopecia areata
HCs	Healthy controls
ECG	Electrocardiography
BMI	Body mass
SALT	Severity of Alopecia Tool
QTc	QT interval
AS	Arterial stiffness
AIx	Augmentation index
PWV	Pulse wave velocity
