# Supplementary material for: Evaluation of Anthropometric Measurements, Arterial Stiffness and ECG Parameters in Alopecia Areata Patients
Source: Medicina (Kaunas). 2025 Nov 28;61(12):2122. doi: 10.3390/medicina61122122 (PMC12734405; doi:10.3390/medicina61122122)
Supplement: Supplementary file 1 [file medicina-61-02122-s001.zip › medicina-4007566-supplementary.pdf]

Supplementary Materials

Figure S1. The power analysis performed for the study

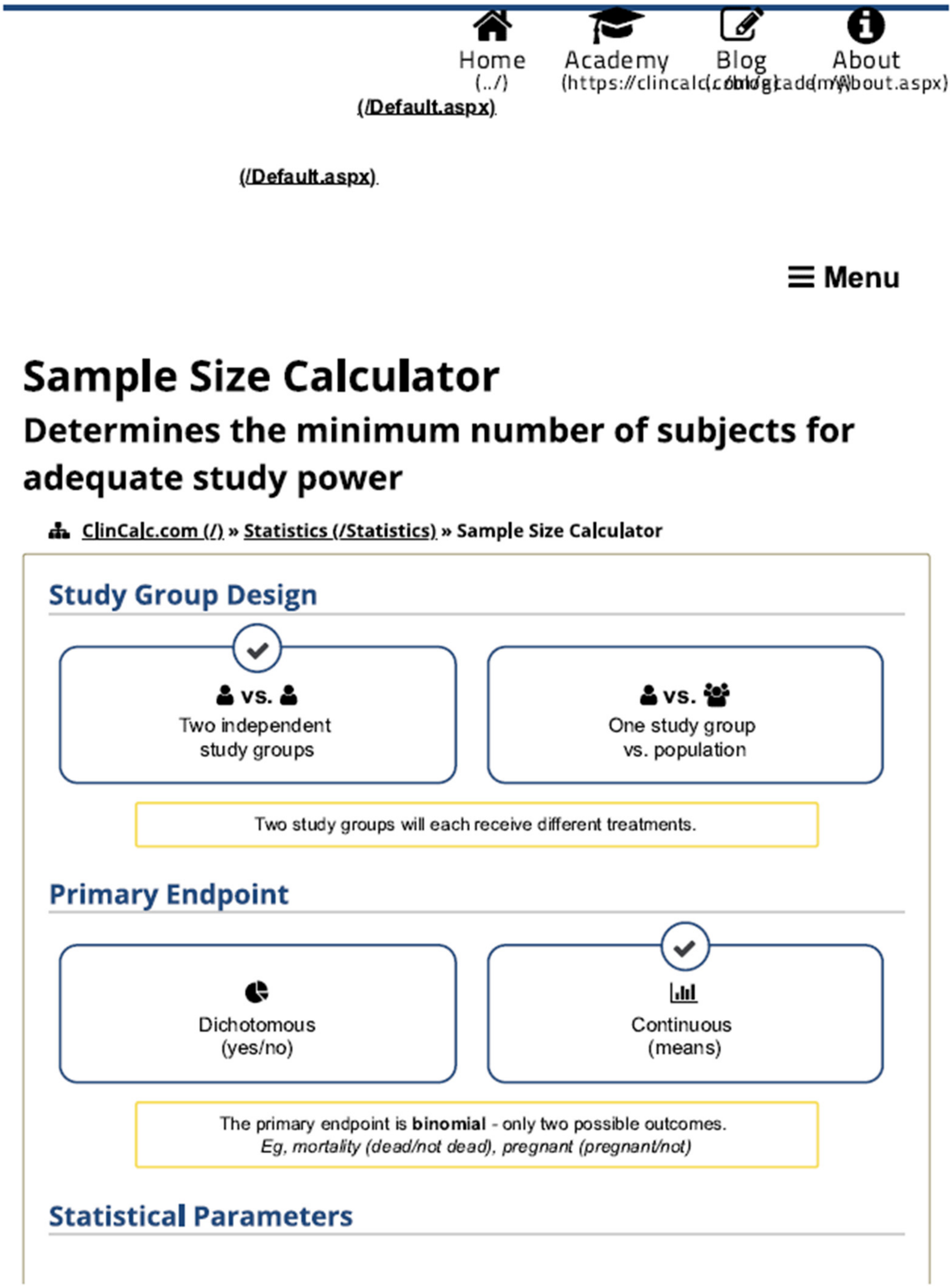

Anticipated Means

Group 1 ? 138.5 ± 67

Group 2 ? 100.5

Mean

Enrollment ratio ? 1

Type I/II Error Rate

Alpha ? 0.05

Power ? 80%

Reset Calculate

RESULTS

Continuous Endpoint, Two Independent Sample Study

Sample Size	
Group 1	49
Group 2	49
**Total**	**98**

Study Parameters	
Mean, group 1	138.5
Mean, group 2	100.5
Alpha	0.05
Beta	0.2

Power	0.8

View Power Calculations

$$k = \frac{n_2}{n_1} = 1$$

$$n_1 = \frac{(\sigma_1^2 + \sigma_2^2/K)(z_{1-\alpha/2} + z_{1-\beta})^2}{\Delta^2}$$

$$n_1 = \frac{(67^2 + 67^2/1)(1.96 + 0.84)^2}{38^2}$$

$$n_1 = 49$$

$$n_2 = K * n_1 = 49$$

$\Delta = |\mu_2 - \mu_1|$ = absolute difference between two means
σ_1, σ_2 = variance of mean #1 and #2
n_1 = sample size for group #1
n_2 = sample size for group #2
α = probability of type I error (usually 0.05)
β = probability of type II error (usually 0.2)
z = critical Z value for a given α or β
k = ratio of sample size for group #2 to group #1

About This Calculator

This calculator uses a number of different equations to determine the minimum number of subjects that need to be enrolled in a study in order to have sufficient statistical power to detect a treatment effect.[1]

Before a study is conducted, investigators need to determine how many subjects should be included. By enrolling too few subjects, a study may not have enough statistical power to detect a difference (type II error). Enrolling too many patients can be unnecessarily costly or time-consuming.

Generally speaking, statistical power is determined by the following variables:

- **Baseline Incidence:** If an outcome occurs infrequently, many more patients are needed in order to detect a difference.
- **Population Variance:** The higher the variance (standard deviation), the more patients are needed to demonstrate a difference.
- **Treatment Effect Size:** If the difference between two treatments is small, more patients will be required to detect a difference.

Table S1. Smoking status among AA patients and controls

**Group**	**Non-smokers (n)**	**Smokers (n)**	**Total (n)**
**Controls**	39	11	50
**AA patients**	31	19	50
**Total (n)**	70	30	100

Statistical test: Pearson Chi-square = 3.048, df = 1, p = 0.081

Table S2. Correlation Between SALT Score and Anthropometric Measurements

**Parameter**	**Correlation Coefficient (r)**	**p-value**
**Height (cm)**	-0.174	0.227
**Weight (kg)**	-0.223	0.120
**Waist circumference (cm)**	-0.190	0.185
**Hip circumference (cm)**	-0.128	0.377
**Body fat percentage (%)**	-0.123	0.395
**Fat mass (kg)**	-0.212	0.139
**Muscle mass (kg)**	-0.194	0.178
**Total body water (kg)**	-0.181	0.210
**Total body water percentage (%)**	0.133	0.355
**Bone mass (kg)**	-0.200	0.163
**Visceral fat level**	**-0.345**	**0.014**
**Body mass index (BMI) (kg/m^2)**	-0.205	0.154

Table S3. Arterial Stiffness Findings of the Patients According to Disease Severity

Parameter	Mild Disease	Moderate and Severe Disease	p-value
**Mean systolic blood pressure (mmHg)**	Mean rank: 26.77	Mean rank: 22.53	0.346
**Mean diastolic blood pressure (mmHg)**	Mean rank: 27.81	Mean rank: 20.10	0.086
**Mean arterial pressure (mmHg)**	Mean rank: 27.50	Mean rank: 20.83	0.138
**Mean pulse pressure (mmHg)**	Mean rank: 24.31	Mean rank: 28.27	0.379
**Mean pulse wave velocity (PWV, m/s)**	Mean rank: 27.46	Mean rank: 20.93	0.146
**Mean augmentation index (AIx)**	21.11 ± 9.57	18.57 ± 12.52	0.438*

*Independent samples t-test was used for AIx comparison; all other variables were analyzed using the Mann–Whitney U test.

Table S4: ECG Findings of the Patients According to Disease Severity

Parameter	Mild Disease	Moderate & Severe Disease	p-value
**Heart rate (beats/min)**	Mean rank: 27.21	Mean rank: 21.50	0.204
**QRS duration (ms)**	87.54 ± 11.67	92.53 ± 9.51	0.151
**QT interval (ms)**	363.94 ± 29.71	363.67 ± 27.47	0.976
**QTc interval (ms)**	Mean rank: 27.69	Mean rank: 20.40	0.105
**PR interval (ms)**	136.49 ± 17.10	137.73 ± 25.52	0.840
**P wave duration (ms)**	103.49 ± 22.53	108.53 ± 26.93	0.497
**P wave amplitude (mV)**	Mean rank: 25.07	Mean rank: 26.50	0.617
**T wave amplitude (mV)**	Mean rank: 23.99	Mean rank: 29.03	0.253
**T peak-to-end interval (ms)**	Mean rank: 23.51	Mean rank: 30.13	0.134
**P wave dispersion (ms)**	Mean rank: 26.77	Mean rank: 22.53	0.316
**QT wave dispersion (ms)**	Mean rank: 25.90	Mean rank: 24.57	0.758

Table S5. Multivariate regression analysis of body fat percentage (%)

Variable	B	Std Error	95% CI	P value
**(Constant)**	-16·893	4·170	-25·175 to -8·610	<0·001
**Alopecia areata**	-1·819	0·769	-3·346 to -0·292	0·020
**Age**	0·164	0·051	0·061 to 0·266	0·002
**Body mass index**	1·550	0·129	1·294 to 1·806	<0·001
**Smoking**	-0·025	0·072	-0·168 to 0·118	0·731
**Sex**	-11·671	0·844	-13·347 to -9·994	<0·001
**Mean systolic pressure**	0·018	0·048	-0·077 to 0·112	0·714
**Mean diastolic pressure**	0·023	0·054	-0·084 to 0·130	0·670

Table S6. Multivariate regression analysis of body fat mass (kg)

Variable	B	Std Error	95% CI	P value
**(Constant)**	-27·451	3·030	-33·469 to -21·433	<0·001
**Alopecia areata**	-1·572	0·559	-2·682 to -0·463	0·006
**Age**	0·055	0·037	-0·019 to 0·130	0·141
**Body mass index**	1·786	0·093	1·600 to 1·971	<0·001
**Smoking**	0·005	0·052	-0·099 to 0·109	0·929
**Sex**	-5·844	0·613	-7·061 to -4·626	<0·001
**Mean systolic pressure**	-0·009	0·035	-0·078 to 0·060	0·800
**Mean diastolic pressure**	0·046	0·039	-0·032 to 0·123	0·247

Table S7. Multivariate regression analysis of visceral fat level

Variable	B	Std Error	95% CI	P value
**(Constant)**	-12.988	1.805	-16.572 to -9.403	<0.001
**Alopecia areata**	-0.283	0.333	-0.944 to 0.378	0.398
**Age**	0.144	0.022	0.100 to 0.189	<0.001
**Body mass index**	0.478	0.056	0.367 to 0.588	<0.001
**Smoking**	0.022	0.031	-0.040 to 0.084	0.478
**Sex**	1.179	0.365	0.454 to 1.904	0.002
**Mean systolic pressure**	-0.002	0.021	-0.043 to 0.039	0.905
**Mean diastolic pressure**	0.017	0.023	-0.029 to 0.063	0.464

Table S8. Multivariate regression analysis of waist circumference (cm)

Variable	B	Std Error	95% CI	P value
**(Constant)**	22.078	5.890	10.379 to 33.746	<0.001
**Alopecia areata**	-0.542	1.086	-2.699 to 1.615	0.619
**Age**	0.116	0.073	-0.028 to 0.261	0.112
**Body mass index**	2.132	0.182	1.7771 to 2.493	<0.001
**Smoking**	0.088	0.102	-0.114 to 0.291	0.388
**Sex**	8.057	1.192	5.689 to 10.424	<0.001
**Mean systolic pressure**	-0.003	0.067	-0.137 to 0.131	0.969
**Mean diastolic pressure**	0.058	0.076	-0.093 to 0.209	0.445

Table S9. Multivariate regression analysis of mean pulse wave velocity (PWV, m/s)

Variable	B	Std Error	95% CI	P value
**(Constant)**	-0·615	0·189	-0·990 to -0·239	0·002
**Alopecia areata**	0·057	0·035	-0·012 to 0·126	0·107
**Age**	0·061	0·002	0·057 to 0·066	<0·001
**Body mass index**	-0·003	0·006	-0·014 to 0·009	0·637
**Smoking**	0·002	0·003	-0·005 to 0·008	0·559
**Sex**	0·036	0·038	-0·040 to 0·112	0·348
**Mean systolic pressure**	0·036	0·002	0·032 to 0·041	<0·001
**Mean diastolic pressure**	-0·003	0·002	-0·007 to 0·002	0·303

Table S10. Multivariate regression analysis of mean augmentation index (AIx)

Variable	B	Std Error	95% CI	P value
**(Constant)**	2·227	9·680	-16·998 to 21·453	0·819
**Alopecia areata**	2·024	1·785	-1·521 to 5·568	0·260
**Age**	-0·078	0·119	-0·315 to 0·159	0·515
**Body mass index**	-0·341	0·299	-0·934 to 0·252	0·256
**Smoking**	0·240	0·167	-0·092 to 0·573	0·155
**Sex**	-7·316	1·959	-11·207 to -3·426	<0·001
**Mean systolic pressure**	0·151	0·111	-0·069 to 0·372	0·175
**Mean diastolic pressure**	0·179	0·125	-0·069 to 0·427	0·154

Table S11. Multivariate regression analysis of QTc interval

Variable	B	Std Error	95% CI	P value
**(Constant)**	395·128	51·589	312·529 to 477·727	<0·001
**Alopecia areata**	-10·675	7·668	-25·905 to 4·555	0·167
**Age**	-0·040	0·512	-1·057 to 0·978	0·939
**Body mass index**	2·537	1·283	-0·011 to 5·085	0·051
**Smoking**	0·521	0·719	-0·908 to 1·950	0·471
**Sex**	-26·352	8·417	-43·069 to -9·636	0·002
**Mean systolic pressure**	-0·524	0·476	-1·470 to 0·421	0·274
**Mean diastolic pressure**	0·351	0·536	-0·714 to 1·416	0·515

Table S12. Multivariate regression analysis of T peak-to-end interval

Variable	B	Std Error	95% CI	P value
**(Constant)**	75·187	7·582	60·128 to 90·246	<0·001
**Alopecia areata**	-1·928	1·398	-4·705 to 0·848	0·171
**Age**	-0·044	0·093	-0·230 to 0·141	0·635
**Body mass index**	0·182	0·234	-0·282 to 0·647	0·438
**Smoking**	-0·135	0·131	-0·396 to 0·125	0·306
**Sex**	4·607	1·535	1·559 to 7·655	0·003
**Mean systolic pressure**	-0·093	0·087	-0·265 to 0·080	0·288
**Mean diastolic pressure**	-0·005	0·098	-0·200 to 0·189	0·956

Table S13. Multivariate regression analysis of T peak-to-interval / QTc interval

Variable	B	Std Error	95% CI	P value
**(Constant)**	0·187	0·075	0·038 to 0·337	0·015
**Alopecia areata**	0·012	0·014	-0·015 to 0·040	0·372
**Age**	<0·001	0·001	-0·002 to 0·002	0·762
**Body mass index**	-0·004	0·002	-0·008 to 0·001	0·133
**Smoking**	<0·001	0·001	-0·003 to 0·002	0·714
**Sex**	0·035	0·015	0·005 to 0·065	0·025
**Mean systolic pressure**	0·001	0·001	-0·001 to 0·003	0·291
**Mean diastolic pressure**	-0·001	0·001	-0·003 to 0·001	0·465
